# The relationship between executive functions and fluid intelligence in euthymic Bipolar Disorder patients

**DOI:** 10.1016/j.psychres.2017.07.066

**Published:** 2017-11

**Authors:** Belén Goitia, Facundo Manes, Teresa Torralva, Mariano Sigman, John Duncan, Marcelo Cetkovich, María Roca

**Affiliations:** a Laboratory of Neuropsychological Research, Institute of Translational and Cognitive Neuroscience (INCyT), INECO Foundation, Favaloro University, Buenos Aires, Argentina; National Scientific and Technical Research Council (CONICET), José Andrés Pacheco de Melo 1854, C1126AAB Buenos Aires, Argentina; b Laboratory of Neuroscience, Torcuato Di Tella University, Av Pres. Figueroa Alcorta 7350, C1428BCW Buenos Aires, Argentina; c National Scientific and Technical Research Council (CONICET), Godoy Cruz 2290, C1425FQB Buenos Aires, Argentina; d MRC Cognition and Brain Sciences Unit, 15 Chaucer Rd, Cambridge CB2 7EF, UK; e Department of Experimental Psychology, University of Oxford, Tinbergen Building, 9 South Parks Road, OX1 3UD Oxford, UK

**Keywords:** Spearman's *g*, Frontal deficits, Multitasking, Theory of mind

## Abstract

Distinct cognitive deficits have been described in Bipolar disorder (BD), including executive impairments, commonly attributed to frontal dysfunction. However, recent attention has been paid to the heterogeneity of cognitive functioning in this population, suggesting that the executive deficits observed in BD might be due to a loss in fluid intelligence (*g*). Following our previous line of investigation in multiple neurological and psychiatric conditions we aimed at determining the role of *g* in frontal deficits in BD. Euthymic BD patients (*n* = 51) and healthy controls (*n* = 37) were assessed with Wisconsin Card Sorting Test (WCST), Verbal Fluency, Trail Making Test B (TMTB), a multitasking test, and a theory of mind test. A general cognitive battery was used to derive a measure of *g*. As in other neuropsychiatric conditions, significant patient-control differences in WCST, Verbal Fluency and TMTB were removed when *g* was introduced as a covariate. Deficits remained significant in the multitasking test. We suggest that neuropsychological assessment in BD should include tests of general intelligence, together with one or more specific tasks that allow for the assessment of residual frontal deficits, putatively associated with anterior frontal functioning.

## Highlights

• We aimed at determining the role of Spearman's *g* in frontal deficits found in BD.
• Deficits in classical executive tests were explained by taking *g* as a covariate.
• Deficits in a multitasking test remained after introducing *g* as a covariate.

## 1 Introduction

Cognitive dysfunction has been widely described in patients with Bipolar Disorder (BD), even during periods of euthymia (Torres et al., 2007, Bora et al., 2009, Bourne et al., 2013). Impairments have been described particularly in executive functions (Arts et al., 2008, Balanzá-Martínez et al., 2010, Kurtz and Gerraty, 2009, Malhi et al., 2007, Seidman et al., 2002, Torres et al., 2007, Zubieta et al., 2001), suggesting that the frontal lobe plays a significant part in this pathology, which is coherent with neuroanatomical findings in this population (Chen et al., 2011, Houenou et al., 2011, Kempton et al., 2008, Strakowski et al., 2012).

Besides the role in executive functions, the frontal lobe has been proposed as a major neural substrate of fluid intelligence (g). The concept of g was introduced by Charles Spearman's g (Spearman, 1904, 1927) to explain universal positive correlations between different cognitive tasks. Spearman proposed that a common g factor contributes to success in all cognitive activities and is best measured by tests of fluid intelligence, which reflects the ability for abstract thought and reasoning, in contrast with ‘crystallized intelligence’ (Cattell, 1971), which depends on prior knowledge and educational achievement (*e.g.* vocabulary). Particularly, lesions in lateral and dorsomedial frontal regions impair the performance in fluid intelligence tests (Duncan et al., 1995, Woolgar et al., 2010) and similar regions are active in functional imaging studies of fluid intelligence test performance (Bishop et al., 2008, Duncan et al., 2000, Esposito et al., 1999, Prabhakaran et al., 1997).

The parallel role of the frontal lobe both in fluid intelligence –which is positively correlated with all tasks– and in executive functions raises the question of how well executive deficits are explained by a fluid intelligence loss. In this regard, multiple studies have shown that fluid intelligence deficits are responsible for the deficits observed in classical executive tests in different neurological and psychiatric conditions. Data from different clinical groups, including patients with frontal lobe lesions (Roca et al., 2010), Parkinson's disease (Roca et al., 2012), Frontotemporal Dementia (Roca et al., 2013), and Schizophrenia (Roca et al., 2014), have consistently shown that after factoring out the effects of *g*, no differences between patients and controls remain for classical executive tests, such as the Wisconsin Card Sorting Test (WCST), Verbal Fluency, and Trail Making Test B (TMTB). On the contrary, other frontal tests, including tests of multitasking and of social cognition, are not explained by differences in *g.* This discordance has been attributed to the fact that those multitasking and social cognition tasks reflect anterior prefrontal deficits rather than the dorsolateral deficits associated with *g* and classical executive deficits (Roca et al., 2010).

The aforementioned findings are not of unimportance to BD research. As it has been mentioned, deficits in classical executive tests are commonly described in BD (Arts et al., 2008, Balanzá-Martínez et al., 2010, Kurtz and Gerraty, 2009, Malhi et al., 2007, Seidman et al., 2002, Torres et al., 2007, Zubieta et al., 2001). Also, multitasking and theory of mind deficits have been described in this population (Bora et al., 2005; Ibáñez et al., 2012; Laloyaux et al., 2013). On this point, it has recently been suggested that the global cognitive impairment observed in some BD patients might be due to deficits in fluid intelligence (Bora et al., 2016) and the disregard of such variable could be a source of error in cluster analytic studies in this population. Even more, since ventral rather than dorsal frontal impairment has been proposed as a marker of BD (Frangou et al., 2005, Bora et al., 2009), the analysis of *g* might allow to differentiate executive deficits and exceeding anterior deficits, becoming fundamental to the understanding of the neuropsychology of BD.

In the present study we aimed at investigating the role of *g* in various frontal deficits in BD. Euthymic BD patients and healthy controls were assessed with WCST, Verbal Fluency, TMTB, a multitasking test, and a theory of mind test. Conventionally, g can be measured either using a standard psychometric test such as Raven's Matrices (Raven et al., 1988) or merely by averaging performance on a varied battery of tests; these two methods give largely comparable results (Cattell, 1971), and we used the latter method here. In this regards, a general cognitive battery was used to derive a measure of *g*. Based on previous findings, and the theoretical and anatomical approaches mentioned before, we predict that for classical executive tests, impairments in BD will be fully explained by a reduction in *g*. Instead, for multitasking and theory of mind we predict that deficits will remain even after removing the effects of *g*.

## 2 Methods

### 2.1 Experiment 1: classical executive functions

#### 2.1.1 Participants

Fifty-seven patients diagnosed with BD, according to the Diagnostic and Statistical Manual of Mental Disorders (DSM-IV, 1994) criteria were recruited from the INECO Data Base in Buenos Aires, Argentina. This allowed us to include in the study only those patients who showed no comorbidity with other neurological or psychiatric diseases. All clinical information, including disease history and drug therapy was provided by a trained psychiatrist specialized in studying BD for over twenty years (co-author Marcelo Cetkovich). From the 57 patients initially recruited, 6 were excluded from the study since they did not meet the euthymia criteria (they presented either Hamilton Depression Rating Scale > 8 or Young Mania Rating Scale > 6 for at least 8 weeks).

Healthy control volunteers (*n* = 37) were recruited through word of mouth from the same geographical area as the patients and were matched to patients, taking into account the mean and range of age and level of education. Participants were included in the control group if they reported no history of neurological or psychiatric disorders, including traumatic brain injury or substance abuse.

Permission for the study was initially obtained from the local research ethics committee and all participants gave their signed informed consent prior to inclusion. The subjects’ consent was obtained according to the Declaration of Helsinki.

#### 2.1.2 Word accentuation test – Buenos Aires (WAT-BA)

To estimate pre-morbid intelligence we used the WAT-BA (Burin et al., 2000). This test, similar to the National Adult Reading Test (Nelson and Willison, 1991), measures the ability to read 51 irregularly stressed Spanish words. The score was the number of words stressed correctly.

#### 2.1.3 General test battery (GTB)

All participants were assessed with a general test battery used to derive a measure of *g*. This battery included Forward Digit Span task (Wechsler, 1997), Rey auditory verbal learning test (Rey, 1941), Rey complex figure test (Rey, 1941), and Trail Making Test A (Partington and Leiter, 1949). For this set of tests, principal component analysis (PCA) produced a first component accounting for 44.4% of the total variance. Loadings on this component were moderate to high for all tests (range = 0.5–0.8). The *g* score for each participant (*g*GTB) was defined as the score on this first principal component. The PCA also produced a second component that was not further considered, accounting for 24.36% of the total variance.

#### 2.1.4 Classical executive battery

##### 2.1.4.1 Wisconsin card sorting test (WCST) (Nelson, 1976)

For the WCST we used Nelson's modified version of the standard procedure. Cards varying on three basic features –colour, shape and number of items– must be sorted according to each feature in turn. The participant's first sorting choice becomes the correct feature, and once a criterion of six consecutive correct sorts is achieved, the subject is told that the rules have changed, and cards must be sorted according to a new feature. After all three features have been used as sorting criteria, subjects must cycle through them again in the same order as they did before. Each time the feature is changed, the next must be discovered by trial and error. Score was total number of categories achieved.

##### 2.1.4.2 *Verbal fluency* (Benton and Hamsher, 1976)

In verbal fluency tasks, the subject generates as many items as possible from a given category. We used the standard Argentinian phonemic version, asking subjects to generate words beginning with the letter P in a one-minute block. Score was the total number of correct words generated.

##### 2.1.4.3 *Trail making test B (TMTB)* (Partington and Leiter, 1949)

In this test the subject is required to draw lines sequentially connecting 13 numbers and 12 letters distributed on a sheet of paper. Letters and numbers are encircled and must be connected alternately (*e.g.,* 1, A, 2, B, 3, C, etc.). Score was the total time (s) required to complete the task, given a negative sign so that higher scores meant better performance.

### 2.2 Experiment 2: multitasking and theory of mind

#### 2.2.1 Participants

A subsample of 24 patients was recruited for Experiment 2. All patients gave informed consent to participate in this second part of the study. Both patients and controls were assessed with a multitasking and a theory of mind test.

#### 2.2.2 *Hotel task* (Manly et al., 2002, Torralva et al., 2009)

The task comprised five primary activities related to running a hotel (compiling bills, sorting coins for a charity collection, looking up telephone numbers, sorting conference labels, proofreading). The materials needed to perform these activities were arranged on a desk, along with a clock that could be consulted by removing and then replacing a cover. Subjects were told to try at least some of all five activities during a 15 min period, so that, at the end of this period, they would be able to give an estimate of how long each task would take to complete. It was explained that time was not available to actually complete the tasks; the goal instead was to ensure that every task was sampled. Subjects were also asked to remember to open and close the hotel garage doors at specified times (open at 6 min, close at 12 min), using an electronic button. Of the several scores possible for this task, we used time allocation: for each primary task we assumed an optimal allocation of 3 min, and measured the summed total deviation (in seconds) from this optimum. Total deviation was given a negative sign so that higher scores meant better performance.

#### 2.2.3 *Faux pas* (Stone et al., 1998)

In each trial of this test, the subject was read a short, one paragraph story. To reduce working memory load, a written version of the story was also placed in front of the subject. In 10 stories there was a faux pas, involving one person unintentionally saying something hurtful or insulting to another. In the remaining 10 stories there were no faux pas. After each story, the subject was asked whether something inappropriate was said and if so, why it was inappropriate. If the answer was incorrect, an additional memory question was asked to check that basic facts of the story were retained; if they were not, the story was re-examined and all questions repeated. The score was 1 point for each faux pas correctly identified, or non-faux pas correctly rejected.

### 2.3 Statistical analysis

All statistical analyses were performed with IBM SPSS^®^ Statistics 20. Groups were compared through Student's *t*-tests for the following variables: age, education years, WAT-BA, WCST, Fonological fluency, TMTB, Hotel task, Faux Pas. As stated above, PCA analysis was performed to obtain a measure of g from the General Test Battery (gGTB, see Section 2.1.3). After this variable was obtained, groups were compared again, this time taking gGTB as a covariate through an ANCOVA, for the following variables: WCST, Fonological fluency, TMTB, Hotel task, Faux Pas. Effect sizes were calculated as Cohen's *d* and were considered large if *d* > 0.8, moderate if *d* > 0.5 and small if *d* > 0.2.

## 3 Results

### 3.1 Experiment 1: classical executive functions

Clinical and demographical data for all participants included in Experiment 1 are shown in Table 1. Results for this experiment are shown in Table 2. For all three tasks, two-tailed *t*-tests were used to compare patients and controls. As expected, the BD group was significantly impaired on all three tasks: WCST, *t(85)* = −3.3, *p* < 0.001; Verbal Fluency, *t(74)* = −2.4, *p* = 0.021; TMTB, *t(83)* = −2.6, *p* = 0.01. Effect sizes (Cohen's *d*) were moderate for the three tests: WCST, *d* = 0.77; Verbal Fluency, *d* = 0.54; TMTB, *d* = 0.61.

**Table 1:** Clinical and demographical data for experiment 1.

	BD		Controls		*p* (two-tailed Student's *t*-test)
	Mean	S.D.	Mean	S.D.	
Age (years)	52.4	16.4	49.4	16.5	0.388
Education (years)	14.9	3.3	14.6	3.1	0.682
WAT-BA	37.6	6.8	38.4	2.5	0.521
Disease duration (years)	13.5	6.5	–	–	–
Age of onset	52.2	11.1	–	–	–
Depressive episodes	5.6	2.5	–	–	–
Manic episodes	3.9	2.0	–	–	–
Hospitalizations	0.4	0.9	–	–	–

**Table 2:** Patient and control scores, average within-group correlation with *g* calculated from the general test battery (*g*GTB), and significance of group differences for each classical executive function task.

	BD			Controls			Patients *vs.* controls *p*	Average within-group correlations with *g*GTB	Patients *vs.* controls after adjustment for *g*GTB *p*
	*n*	Mean	S.D.	*n*	Mean	S.D.			
*g*GTB	51	−0.38	1.1	37	0.52	0.57	< 0.001	–	–
WCST	50	4.9	1.7	37	5.8	0.4	< 0.001	0.261	0.190
Verbal fluency	39	15.5	4.4	37	17.9	4.5	0.021	0.476	0.835
TMTB	48	−113.8	93.0	37	−71.5	31.6	0.01	0.547	0.294

**Fig. 1:**
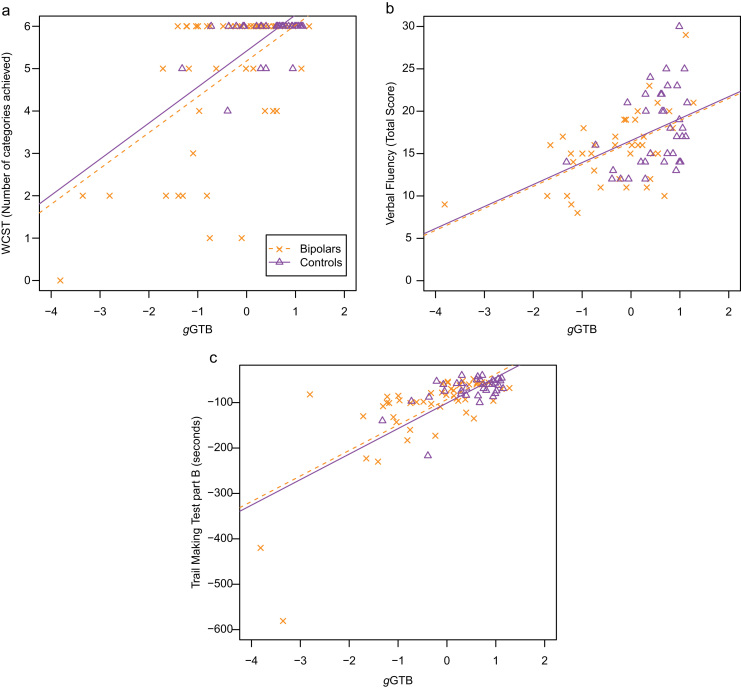
Scatter plots relating performance in (a) the Wisconsin Card Sorting Test (WCST), (b) Verbal Fluency and (c) Trail Making Test part B (TMTB) to *g*GTB for patients with bipolar disorder (squares) and controls (triangles). Regression lines (broken for bipolars and solid for controls) reflect the average within-group association of the variables, as determined by ANCOVA, constrained to have the same slope across groups.

Also as expected, the three executive tests were correlated with *g*GTB. Combining data from patients and controls, average within-group correlations with *g*GTB were *r* = 0.565 for WCST, *r* = 0.470 for Verbal Fluency, and *r* = 0.693 for TMTB (Table 2). Scatter plots in Fig. 1 show that higher *g*GTB was strongly associated with better performance in all three executive tasks.

The scatter plots (Fig. 1) suggest that, for these executive tasks, frontal deficits were entirely explained by fluid intelligence, as there is no apparent effect of group above and beyond the linear regression to *g*GTB. To assess this conclusion, the comparison of patients and controls was repeated following adjustment for *g*GTB as a covariate. For all three tasks, the difference between patients and controls was no longer significant when *g*GTB was taken as a covariate (ANCOVA): for WCST, *F*_*(1,84)*_ = 0.608, *p* = 0.438; for Verbal Fluency, *F*_*(1,73)*_ = 0.044, *p* = 0.835; and for TMTB, *F*_*(1,82)*_ = 0.382, *p* = 0.538; (Table 2).

### 3.2 Experiment 2: multitasking and theory of mind

Clinical and demographical data for all participants in this experiment are shown in Table 3. Again, two-tailed *t*-tests were used to compare patients and controls (*n* = 35). Results are shown in Table 4. The BD group was significantly impaired on both the Hotel task (*t(52)* = −4.29, *p* = < 0.001) and the Faux Pas task (*t(57)* = −3.3, *p* = 0.002). Effect sizes (Cohen's *d*) were large for both tests: Hotel task, *d* = 1.04; Faux Pas task, *d* = 0.82.

**Table 3:** Clinical and demographical data for experiment 2.

	BD		Controls		*p* (two-tailed Student's *t*-test)
	Mean	S.D.	Mean	S.D.	
Age (years)	54.8	17.8	48.8	16.8	0.191
Education (years)	15.0	3.7	14.7	3.2	0.811
WAT-BA	37.1	8.3	38.5	2.5	0.397

**Table 4:** Patient and control scores, average within-group correlation with *g* calculated from the general test battery (*g*GTB), and significance of group differences for each classical executive function task.

	BD			Controls			Patients *vs.* controls *p*	Average within-group correlations with *g*GTB	Patients *vs.* controls after adjustment for *g*GTB *p*
	*n*	Mean	S.D.	*n*	Mean	S.D.			
*g*GTB	24	−0.47	1.24	35	0.32	0.64	0.002	–	–
Hotel task	19	−596.3	339.5	35	−315.7	140.4	< 0.001	0.370	0.002
Faux Pas	24	16.7	3.0	35	18.6	1.3	0.002	0.437	0.055

**Fig. 2:**
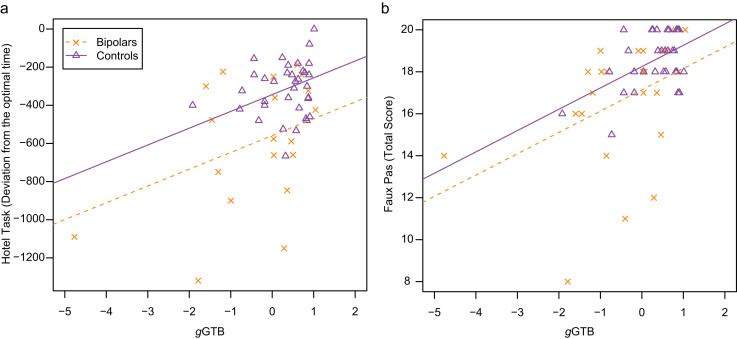
Scatter plots relating performance in (a) the Hotel task, and (b) Faux Pas to *g*GTB for patients with bipolar disorder (squares) and controls (triangles). Regression lines (broken for bipolars and solid for controls) reflect the average within-group association of the variables, as determined by ANCOVA, constrained to have the same slope across groups.

For both tasks, correlations with *g*GTB were positive, showing better performance associated with higher fluid intelligence (Table 4). Scatter plots relating performance to *g*GTB are shown in Fig. 2. Contrary to the results from Experiment 1, these scatter plots suggest some difference between patients and controls even when correcting for the difference in *g*GTB. As before, the groups were compared after taking *g*GTB as a covariate (ANCOVA): for the Hotel task, *F*_*(1,51)*_ = 10.7, *p* = 0.002; for the Faux Pas task, *F*_*(1,56)*_ = 3.8, *p* = 0.055 (Table 4).

## 4 Discussion

Diverse cognitive deficits have been described in Bipolar disorder (BD), including executive impairments and social cognition deficits (Arts et al., 2008, Balanzá-Martínez et al., 2010, Bora et al., 2005, Ibáñez et al., 2012, Kurtz and Gerraty, 2009, Laloyaux et al., 2013, Malhi et al., 2007, Seidman et al., 2002, Torres et al., 2007, Zubieta et al., 2001), which are commonly attributed to frontal dysfunction. Also, it has recently been suggested that the heterogeneity of cognitive functioning in this population might be related to fluid intelligence (*g*). Following our previous line of research we aimed at investigating if a loss in *g* could explain some of those deficits. In order to do so, BD patients and healthy controls were assessed with a range of frontal tests and with a general cognitive battery used to derive a measure of *g*. We then searched for differences between the two groups before and after taking *g* as a covariate.

Consistent with previous findings in other pathologies with frontal involvement (Roca et al., 2014, Roca et al., 2013, Roca et al., 2012, Roca et al., 2010), we observed that once the effects of fluid intelligence are removed, there are no differences between BD patients and controls in classical executive tests (WCST, Verbal Fluency and TMTB), suggesting that a loss of fluid intelligence in BD fully explains deficits in these tests.

Also in agreement with previous findings (Roca et al., 2014, Roca et al., 2013, Roca et al., 2012, Roca et al., 2010), deficits in fluid intelligence could not explain the differences observed between groups in multitasking (Hotel task). Elsewhere we have proposed that, unlike classical executive tests, which may rely heavily on the integrity of the dorsolateral prefrontal cortex, multitasking tests are more dependent on the anterior frontal cortex (Gilbert et al., 2006, Roca et al., 2010).

Regarding theory of mind, even though in other frontal pathologies deficits were not explained by *g* (Roca et al., 2014, Roca et al., 2013, Roca et al., 2012, Roca et al., 2010), in this case our results were not as conclusive. Contrary to what we predicted, the inclusion of *g*GTB as a covariate led to a group effect on the edge of significance (*p* = 0.055). Future research should aim at testing larger numbers of patients to elucidate the true link of *g* to social cognition in this pathology.

The fact that in this population fluid intelligence explained deficits in classical executive tasks but not in multitasking is compatible with the idea that this pathology has a strong anterior prefrontal component that exceeds dorsolateral frontal functioning. Our data is also in concordance with the suggestion that some of the heterogeneity of cognitive deficits in this population could be related to differential fluid intelligence deficits in BD patients (Bora et al., 2016).

The results presented here have important clinical implications. Primarily, as the deficits detected might reflect a general cognitive impairment instead of task-specific deficiencies, it would be advisable to include a measure of general intelligence in order to achieve a proper neuropsychological assessment of BD patients. Focusing only on specific executive function tests could lead to missing important information about BD patients’ cognitive function and consequently to ineffective treatment. On the other hand, our data have strong implications for the clinical use and interpretation of test such as the Wisconsin Card Sorting Test, Verbal Fluency and Trail Making Test in Bipolar Disorder. Largely, the deficits detected in such tests seem not to be related specifically to their particular cognitive content, but instead, they seem to reflect a much more general cognitive loss. Further studies should investigate if this may hold for other popular ‘classical executive’ tests widely used in this population.

In addition to this, other tests should be considered in order to examine residual frontal deficits unrelated to *g* that might be present in the pathology of BD.

Again, from a clinical perspective, our results may have important implications in understanding the differences between psychiatric conditions, such as schizophrenia and BD. Given the overlap in symptomatology (including overlaps in neurocognitive domains) and the fact that both conditions share clinical, epidemiological and etiological characteristics, a debate about the real characteristics of their nosological limits has arisen in the psychiatric community (Craddock et al., 2006, Lichtenstein et al., 2009, Maier et al., 2006). Schizophrenia patients often present cognitive deficits in attention, working memory and other executive functions (Conklin et al., 2000, Goldberg et al., 1993, Heinrichs and Zakzanis, 1998, Park and Holzman, 1992, Sharma and Antonova, 2003), and deficits in classical executive tests are explained by a loss in Spearman's *g* (Roca et al., 2014). Recognizing if there is a differential role of *g* in frontal deficits shown in both pathologies may shed some light on this debate and on the differences of their functional outcome.

Finally, even if spectacular achievements have been made in the comprehension of frontal lobe functioning, the advances in cognitive neuroscience have not yet been translated to the correct understanding of clinical conditions. We believe that our results represent a step forward to the required assembly of experimental neuroscience and clinical neuropsychology in the effort to understand frontal lobe functioning in neurological and psychiatric conditions with frontal involvement such as BD.

## Financial support

This work was supported by Medical Research Council (UK) intramural program MC-A060-5PQ10 and by grants CONICYT/FONDECYT Regular 1130920, PICT 2012-0412*,* CONICET and INECO Foundation.

## Conflicts of interest

None
